# Towards Better CARE for Superficial Fungal Infections: A Consultation Guide for the Community Pharmacy

**DOI:** 10.3390/pharmacy10010029

**Published:** 2022-02-11

**Authors:** Pantira Parinyarux, Wiwat Thavornwattanayong, Cheardchai Soontornpas, Peeranon Rawangnam

**Affiliations:** 1 Faculty of Pharmacy, Payap University, Chiang Mai 50000, Thailand; parinyarux.p@gmail.com; 2 Faculty of Pharmacy, Silpakorn University, Nakorn Pathom 73000, Thailand; 3 Faculty of Pharmaceutical Sciences, Khon Kaen University, Khon Kaen 40002, Thailand; chesoo@kku.ac.th; 4 Community Pharmacist, Bangkok 77110, Prachuap Khiri Khan, Thailand; rawangnam@gmail.com

**Keywords:** superficial fungal infections, pharmacists, consumers, self-care, healthcare partnership, pharmacist consultation, ASEAN region

## Abstract

Superficial fungal infections (SFIs) are among the most common skin diseases worldwide and are common in many parts of Asia. Community pharmacists are well-placed to help identify and manage SFIs. However, effective management may be hindered by a suboptimal consultation process, attributed to the misalignment between consumers’ and pharmacists’ viewpoints. The Fungal CARE (Care, Assess, Recommend, Empower) guide, a patient-centered collaborative framework, was developed to improve pharmacist-led SFI consultations in community pharmacy. A survey on real-world consumer experiences with SFIs provided insights for aligning the Fungal CARE guide with consumer perspectives. To further optimize the guide, community pharmacists were surveyed on their current practice and challenges of managing SFIs, as well as views on the usefulness of the Fungal CARE guide. The pharmacists’ survey indicated that respondents engaged with some but not all of consumers’ top concerns with SFIs, such as emotional and social aspects. Pharmacists identified their greatest challenges as poor compliance with SFI treatment and limited confidence in identifying and/or managing SFIs. Encouragingly, when presented with the Fungal CARE guide, nearly all pharmacists agreed it would be helpful and would use it in practice. Implementing the Fungal CARE guide may help improve pharmacist-led consultations for SFIs and encourage better treatment outcomes.

## 1. Introduction

Superficial fungal infections (SFIs) are among the most common skin diseases worldwide, affecting nearly one billion people annually [1]. Due to the warm and humid climate in many parts of Asia, fungal infections tend to be more prevalent than in drier climates [2]. The etiological landscape and causative organisms are relatively similar across the region; for example, tinea imbricata is endemic to Malaysia, Vietnam, Thailand, and the Philippines [3].

Clinical features of the condition include skin irritation, redness, itching, swelling, and blisters around the affected area [4]. These symptoms, particularly skin itching, peeling, and redness, are commonly experienced by individuals with SFIs and can significantly reduce wellbeing and quality of life [5,6,7]. Moreover, if left untreated, persistent scratching due to itching may accelerate tissue damage and delay healing, potentially leading to secondary infections [8]. Beyond the burden of these physical symptoms, individuals with SFIs commonly experience negative emotions such as feelings of anxiety or stress that may hinder them from participating in social and leisure activities [5,7].

SFIs can be caused by multiple organisms, including dermatophytes, molds, and yeasts. This, along with the similarities between SFI symptoms and those of certain common skin conditions, especially eczema, can make diagnosis and management of SFIs potentially challenging [4]. As such, SFIs are frequently misdiagnosed and inappropriately treated, which may in turn complicate subsequent management and increase the overall burden of care [9,10]. Although controlled trials showed that existing treatments are efficacious when the SFI is correctly diagnosed and treatment guidelines are followed [9], persistent challenges such as poor compliance and infection recurrence remain obstacles to real-world SFI management [11,12,13].

Accurate identification and initiation of appropriate treatment are essential to reduce the burden of SFIs, and community pharmacy services are well-positioned to facilitate this. For consumers in Asia, community or retail pharmacies often serve as an initial point of contact for healthcare because of their convenient and accessible location within communities, extended opening hours, and perceived affordability [14,15,16,17]. Community pharmacies are an important part of the healthcare service ecosystem in Asia; such facilities account for a substantial proportion of the drug retail outlets in many countries, and a relatively large proportion of pharmacists practice in community pharmacy settings in countries including the Philippines, Vietnam, and Thailand [18,19,20].

Although medication dispensing and provision of limited medication advice currently remain the core role of community pharmacists across Asia, their role has been expanding to include additional healthcare services, such as the management of minor ailments, health screening, and health promotion advice [16,21,22,23]. This reflects a move towards improving pharmacy practice standards internationally, in line with the Good Pharmacy Practice standards endorsed by the International Pharmaceutical Federation and the World Health Organization [24]. In the course of providing such additional health services, pharmacists play a large role in promoting self-care by individuals, an increasingly important concept in public health and chronic disease management [25,26,27,28]. As a first touch-point for consumers, community pharmacists can provide initial SFI assessment and follow-up, advise on acute management, self-care, and prevention, and refer individuals for specialist care if necessary. Maximizing the use of pharmacy services for successful disease management in the community would be beneficial, especially during the current coronavirus disease 2019 (COVID-19) pandemic, by helping to reduce the burden on specialist and tertiary care [26,27,28].

To deliver on this vision of effective patient-centered community care, pharmacists need the support of an effective consultation process, especially when engaging with consumers to manage their expectations and concerns about treatment outcomes. To address this unmet need, the Fungal CARE guide, a practice resource for community pharmacists managing SFIs, is introduced. This paper describes the concept and development of the Fungal CARE guide. It is adapted from a four-step CARE (Categorize, Assess, Recommend, and Empower) consultation framework, first proposed to support integrative and personalized nutrition counselling in primary care settings (community and hospital pharmacies) [29]. The CARE consultation framework provides community pharmacists with a structured yet collaborative patient-centered approach to improve the quality of care, similar to other established models such as the Pharmacists’ Patient Care Process in the USA [30].

To optimize the CARE framework for guiding SFI management, the authors drew on real-world insights from two surveys on (i) consumers’ experiences with SFIs and (ii) practicing pharmacists’ perspectives on SFI management and challenges. They also sought feedback from community pharmacists on the potential applicability and relevance of the Fungal CARE guide. In this paper, both consumer and pharmacist perspectives are presented and were used to guide the adaptation of the Fungal CARE guide as a consultation framework for community pharmacists.

## 2. Real-Life Experiences and Concerns of Consumers with SFIs

**Figure 1:**
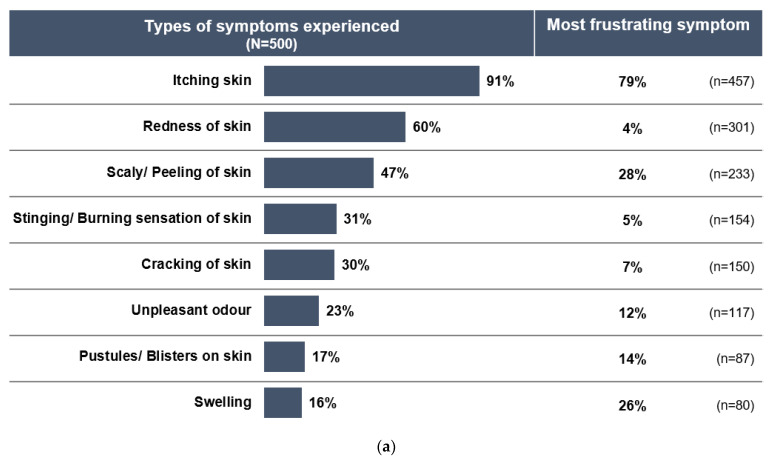

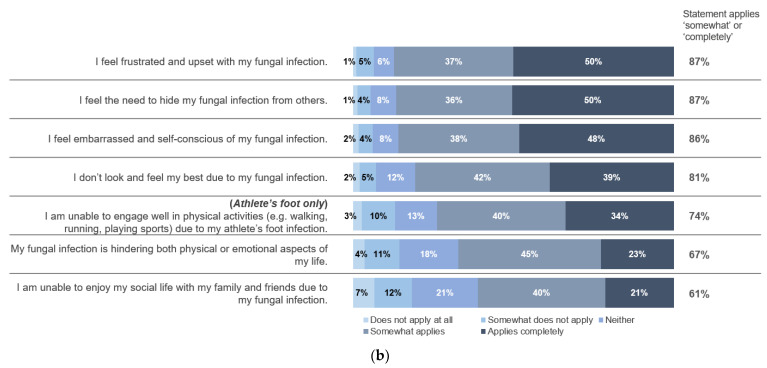
Consumer experiences with SFIs. (**a**) Types of symptoms experienced by consumers with SFIs. (**b**) Feelings experienced by consumers with SFIs during fungal infection.

An online survey was conducted from 31 March to 17 June 2020 to retrieve usage and satisfaction data from consumers in the Philippines who had used a topical treatment (clotrimazole) in the last 12 months to treat their most recent SFI. Respondents were recruited from an online consumer panel and were invited to participate in the online survey via a web link that directed them to a screening questionnaire. Respondents who met the screening criteria (male or female, aged 20–45 years, residing in any region of the Philippines) were invited to complete the full survey, with anonymized responses collected via a dedicated online platform. Further details are provided in Supplementary File S1. A total of 500 respondents were analyzed. The majority of respondents (56%) were from Metro Manila (Luzon, capital region), 26% from Balance Luzon (Luzon, outside of the capital region), 11% from Mindanao, and 7% from Visayas. The age distribution was relatively uniform across the range of 20–45 years: 20–25 years (26%), 26–30 years (25%), 31–35 (24%), and 36–45 (25%), and the average age was 30.9 years. The survey showed that almost 90% of respondents had previously suffered from SFIs. The most recent SFI was ringworm (29% of respondents), followed by white spots (26%), jock itch (23%), and athlete’s foot (22%). Skin itching was by far the most common symptom reported (91% of consumers), followed by skin redness (60%) and scaly or peeling skin (47%) (Figure 1a). Notably, 79% of consumers also highlighted skin itching as the symptom with which they felt most frustrated and rated the severity as moderate to severe on average (7.4 on a scale from 1—no itch to 10—worst possible itch).

Besides physical symptoms, more than 85% of respondents reported frustration and embarrassment about their diagnosis and felt the need to hide their condition. In addition, 61% reported that their fungal infection hindered them from enjoying social activities. Among those with athlete’s foot, 74% of respondents felt restricted from engaging in physical activities such as walking, running, and playing sports due to their infection (Figure 1b).

On average, respondents reported that their infections lasted around two weeks (mean 15.5 days). After applying the topical antifungal medication (clotrimazole), 79% experienced symptom relief after 0–3 days, 20% after 4–7 days, and 1% reported no symptom relief after 7 days. Notably, treatment compliance appeared low: 72% of respondents reported discontinuing medication use once they experienced symptom relief, despite the treatment duration recommended on product labels (typically 1–4 weeks, depending on the type of SFI). This finding indicates that it is common for many individuals with SFIs to prematurely discontinue treatment, which may increase the chance of treatment failure and recurrence.

The survey results clearly show the negative impact that SFIs have on individuals. Beyond debilitating symptoms such as moderate–severe itching, there is considerable emotional and social impact. Premature discontinuation of treatment also remains a key concern. Therefore, it is important for pharmacists to be aware of these aspects when counselling consumers with SFIs to optimize communication and information exchange and ultimately the quality of care [31].

## 3. Current Practice, Perceptions, and Challenges among Pharmacists

An online pilot survey was conducted from June−July 2021 among pharmacists in Thailand to gather data on current challenges and practices in SFI management and ascertain how consultation aids such as the Fungal CARE guide could be best utilized in community pharmacy settings. The survey criteria included pharmacists practicing in any professional roles, settings or healthcare facility types, in any region of the country. Respondents were invited to participate in the survey via invitations through email, online pharmacist network groups, social media, and quick response (QR) codes displayed in pharmacies, which directed them to the online survey website. Before completing the survey, respondents provided consent for their responses to be recorded anonymously and analyzed in aggregate. Further details are provided in Supplementary File S2. A total of 104 responses from pharmacists were analyzed. Of these, most respondents were community pharmacists (85%), practiced in a private setting (96%), and >70% of the respondents had been practicing for more than five years. The majority of respondents (45%) practiced in the Bangkok area, although other regions of the country were well represented (North: 27%; South: 19%; Northeast: 9%). More than half of the pharmacists (53%) reported five or more SFI-related consultations per week and estimated that most of these consultations were with working adults. The top reason for SFI-related consultations, cited by 77% of pharmacists, was to obtain medication to relieve symptoms such as skin itching or redness. Most pharmacists observed that consumers generally did not have a treatment in mind when visiting the pharmacies, but sought advice to understand their condition better and use medications appropriately.

Pharmacists relied primarily on visual lesion assessment (91%) and symptoms presented (58%) to identify SFIs and rarely used diagnostic tools or tests (<5%). When advising on SFI management, most pharmacists reported relying on treatment guidelines (82%) and their own clinical experience (60%). More than half of the respondents (53%) based their treatment recommendations on the options available in their practice settings, and 28% reported taking patient preference into consideration.

**Figure 2:**
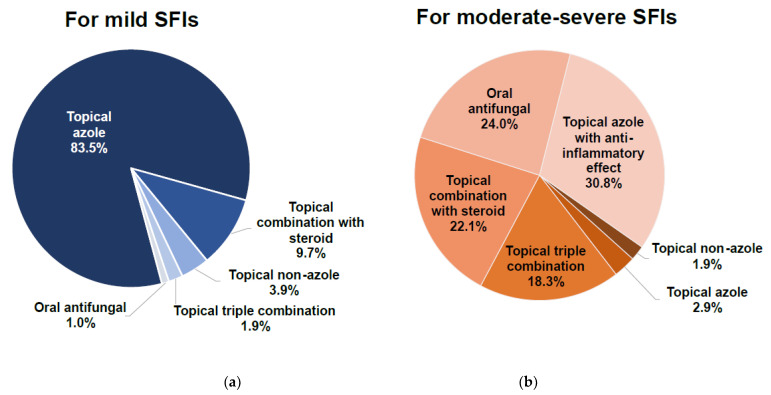

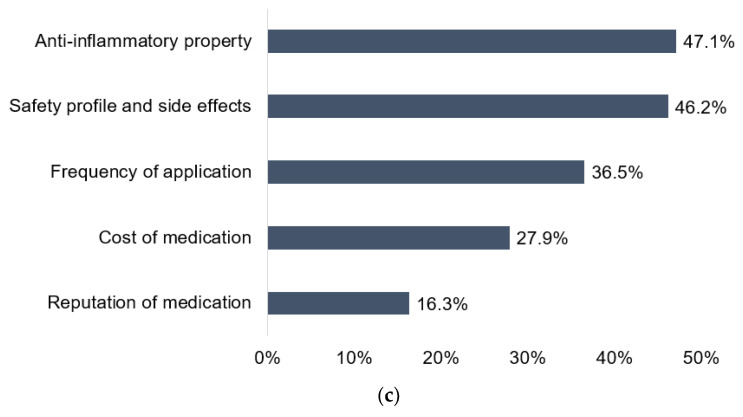
Selection of topical azoles for treatment of superficial fungal infections. (**a**) Consideration for mild infections. (**b**) Considerations for moderate to severe infections. (**c**) Overall considerations.

Available SFI treatment options include a range of azole or non-azole topical preparations, topical antifungal-corticosteroid combination preparations, and oral antifungal preparations. Based on our survey, most pharmacists preferred to recommend topical azoles for treating SFIs, whether mild or moderate/severe. For mild infections, 84% of pharmacists would recommend topical azoles without additional properties (Figure 2a). For moderate to severe infections, pharmacists preferred to recommend a topical azole with anti-inflammatory effects (31%) over an oral antifungal (24%) or a topical azole combined with steroids (22%) (Figure 2b). In contrast, 84% of pharmacists noted a preference among some consumers for topical azole combinations with steroids. When choosing among topical azoles, pharmacists regarded anti-inflammatory properties (47%), safety profile (46%), and frequency of application (37%) as their top overall considerations (Figure 2c)

**Figure 3:**
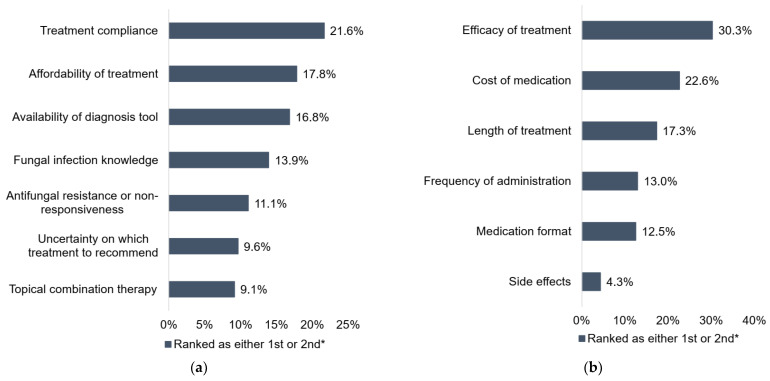
Challenges of managing superficial fungal infections. (**a**) Top challenges identified by pharmacists. (**b**) Top factors affecting treatment compliance. * Indicates percentage of respondents that ranked the option in either the first or second place (1—most important or relevant; 6 or 7—least important or relevant).

Overall, pharmacists identified treatment compliance as one of their main challenges in managing SFIs, followed by treatment affordability considerations, availability of diagnostic tools and limited knowledge of SFIs (Figure 3a). Among various factors affecting treatment compliance, pharmacists identified treatment efficacy, cost of medication, and length of treatment as the most important factors (Figure 3b). Besides these specific challenges articulated by the pharmacists, the survey responses suggested additional areas for improvement: up to 40% of pharmacists felt uncertain when it came to identifying SFIs, and 25% when managing SFIs.

Together, the consumer and pharmacist survey results highlight the presence of well-known issues in SFI management in both settings, most notably treatment compliance. Importantly, the findings also point to some misalignment in consumers’ and pharmacists’ respective viewpoints on two areas: first, the understanding of treatment compliance and its importance for successful treatment; second, the extent of the impact SFIs have on an individual’s emotional as well as physical wellbeing. By applying a structured and patient-centered approach right from the first consultation, pharmacists can work with the consumers to systematically address these and other challenges in SFI management. This approach will also be advantageous to make the best use of limited consultation time and potentially improve the quality and value of care delivered.

## 4. Improving Pharmacist-Consumer Consultations with the Fungal CARE Guide

**Figure 4:**
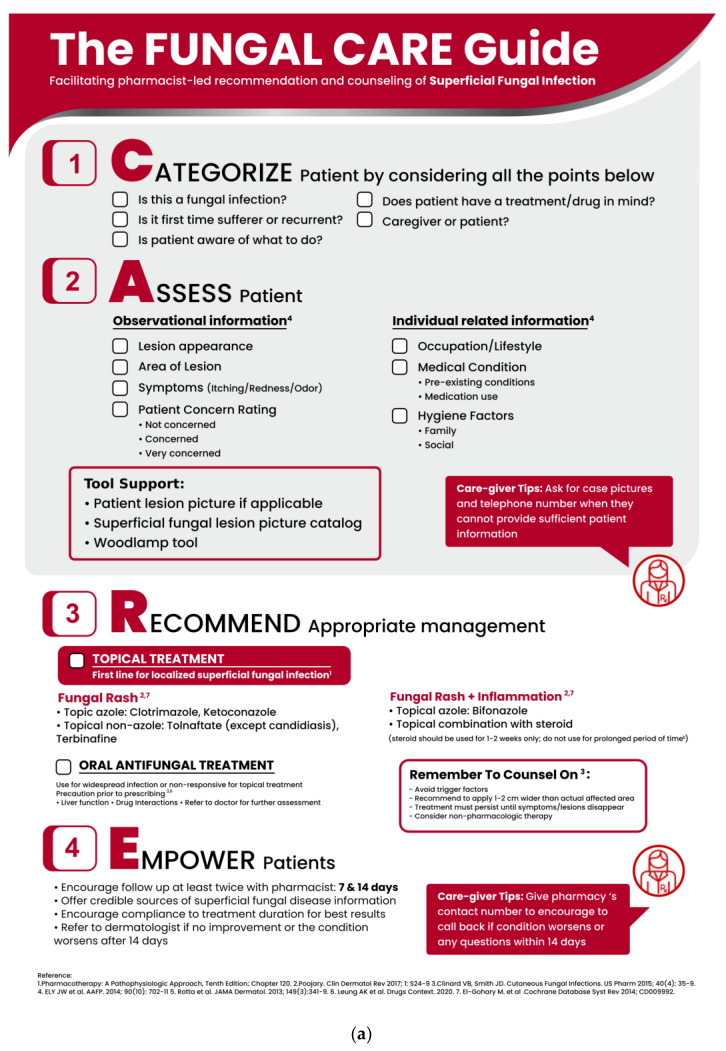

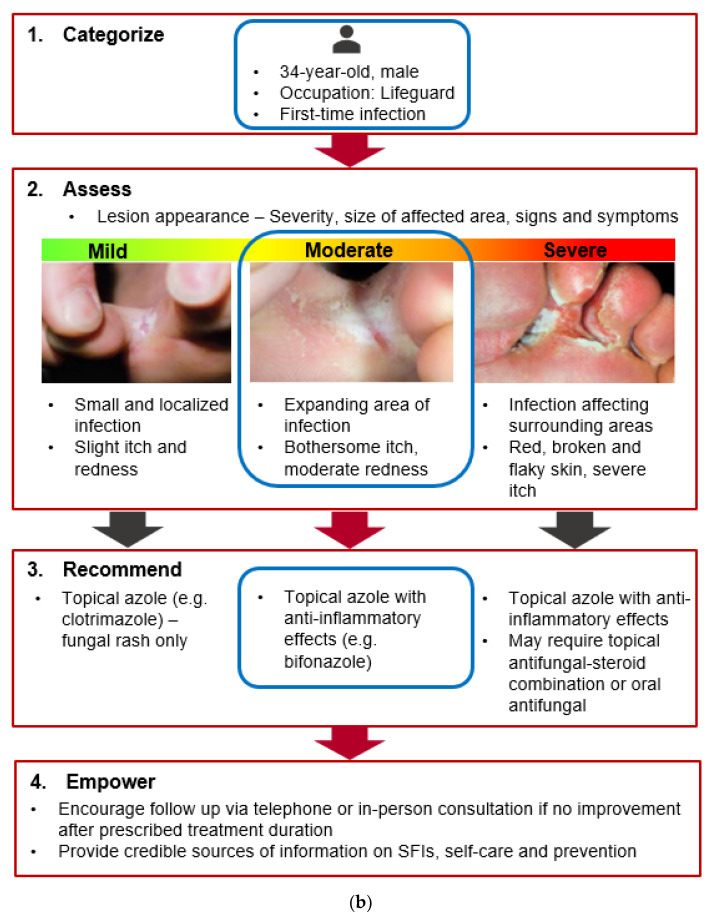
The Fungal CARE Guide. (**a**) Four-step consultation framework to support the management of superficial fungal infections (SFI) in the community. (**b**) Hypothetical example showing application of the guide for SFI management.

The CARE framework was originally developed to support pharmacists in patient-centered, personalized counselling on nutrition and dietary supplements [29]. Due to the practicality and versatility of the CARE framework, it was feasible to adapt it to create the Fungal CARE guide to support consultations for SFIs in pharmacies (Figure 4a). The guide focuses especially on types of fungal infections that can be treated with topical medications, rather than systemic treatments.

The first step of the Fungal CARE guide, *Categorize*, prompts pharmacists to elicit relevant information about the consumer and the condition that led them to seek attention (Step 1, Figure 4a). If a fungal infection is suspected, pharmacists should ascertain whether it is a first-time or recurrent infection. If the consumer reports a recurrent infection, pharmacists should ask open-ended questions on how they have been using their medications and any issues faced before deciding on treatment recommendations. In the hypothetical case example (Figure 4b), the pharmacist could establish whether or not the individual has previously had a fungal infection and ask whether he has any type of drug treatment in mind.

The second step, *Assess*, ensures that the SFI is accurately identified, so that appropriate treatment can be recommended. This starts with a careful visual assessment of the lesion(s), wherever feasible, and eliciting information on symptoms such as itching or redness to categorize the condition as either mild, moderate or severe (Step 2, Figure 4a). If visual assessment is not possible (e.g., the individual with the SFI is not present, or due to the anatomical location of the lesions), pharmacists can rely on photographs or video images to assess the severity and extent of the infection. If available, tools and resources such as an SFI lesion picture catalogue or Wood’s lamp do help in identifying the SFI. Questions about medications, allergies or other medical conditions, occupational or lifestyle factors, and hygiene routines are also relevant. In the case example, the pharmacist would note that redness and signs of inflammation are present and would ask questions to assess how the individual’s occupation as a lifeguard might affect his ability to comply with treatment (Figure 4b).

The third step, *Recommend*, involves selecting the most appropriate treatment for the consumer’s infection and circumstances. The authors advocate an informed and shared decision-making process based on the assessment done in Steps 1 and 2, with the consumer’s agreement and cooperation. The CARE guide provides a quick reference on commonly used medications for mild, moderate, and severe SFIs, as well as considerations for fungal rash with or without inflammation (Step 3, Figure 4a). Depending on the individual and the characteristics of their SFI, azoles with additional properties may represent a suitable option [12]. If recommending a topical azole, the pharmacist can demonstrate how much topical medication to apply and suggest the best time to apply it to ensure adequate dosing. In the hypothetical case depicted in Figure 4b, given that the individual’s occupation subjects him to prolonged water exposure and that his skin lesions also show signs of inflammation, the pharmacist could recommend a topical azole with anti-inflammatory properties and once-daily application (e.g., bifonazole) to be applied before going to bed. In addition to recommending treatment options, the pharmacist is also encouraged to provide practical advice on appropriate hygiene and self-care practices [32,33].

The last step, *Empower*, emphasizes the active role that consumers can play in positive treatment outcomes by increasing their health literacy and practicing self-care. The consumer survey showed that many consumers were unaware of the recommended treatment duration for SFIs and that premature discontinuation is linked to suboptimal treatment outcomes. Therefore, it is crucial for pharmacists to help consumers improve their health literacy and minimize misinformation [26]. In the example, the pharmacist should strongly encourage the individual to continue applying the medication for the full recommended treatment period while doing his best to maintain good personal hygiene and explain clearly when to return for follow-up or seek specialist referral (if their condition worsens or symptoms persist after completing treatment; Step 4, Figure 4). Pharmacists can provide additional information on SFI-related self-care and prevention and how best to contact a pharmacist if specific questions or issues arise.

The applicability of the Fungal CARE guide was presented and evaluated in the pharmacist survey. Overwhelmingly, the respondents (92%) considered the Fungal CARE guide helpful in guiding the selection of treatment for SFI. Additionally, >95% of the respondents indicated that they intended to use the Fungal CARE guide in their practice.

## 5. Discussion

The results of the consumer and pharmacist surveys together serve to highlight current unmet needs in managing SFIs within the community pharmacy setting. These unmet needs can potentially be addressed by improving pharmacist-consumer consultations with the help of a framework such as the Fungal CARE guide.

A sizable proportion of the surveyed pharmacists expressed uncertainty about their knowledge and confidence in correctly identifying SFIs during consultation and choosing appropriate treatments. Using a systematic framework during consultations, supplemented by quick-reference guides (such as an SFI lesion picture catalogue), could build confidence and help pharmacists apply their knowledge more effectively to correctly identify the SFI based on visuals and elicit comprehensive symptom or history details. The Fungal CARE guide further summarizes key practical considerations for selecting topical treatments, including newer options with additional properties that some pharmacists may be less familiar with.

Treatment compliance was highlighted as a major concern in both the consumer and pharmacist surveys. Especially among pharmacists, this was the top concern, since poor compliance leads to symptom recurrence [34]. However, the consumer survey showed that many individuals were unaware of the importance of completing the prescribed treatment course, with most reporting that they stopped applying their medication after experiencing some relief of symptoms. In the authors’ experience, there are three common reasons for poor compliance related to actual or perceived low treatment efficacy. Firstly, initial misidentification of the consumer’s condition and recommendation of an inappropriate medication might delay recovery or even worsen the condition. It is therefore important for pharmacists to carefully assess the lesion visually and utilize diagnosis aids or tools when required. Second, clear dosage instructions for topical preparations are a challenge, and this can lead to suboptimal or incorrect medication application [35]. Lastly, some consumers may have impractical expectations due to a poor understanding of their condition or how topical antifungal medications work, leading them to conclude prematurely that a treatment was ineffective [36]. The authors identify these as important communication gaps and suggest a systematic approach that community pharmacists can leverage to enhance health literacy among consumers and empower consumers to practice self-care and take charge of their health.

**Figure 5:**
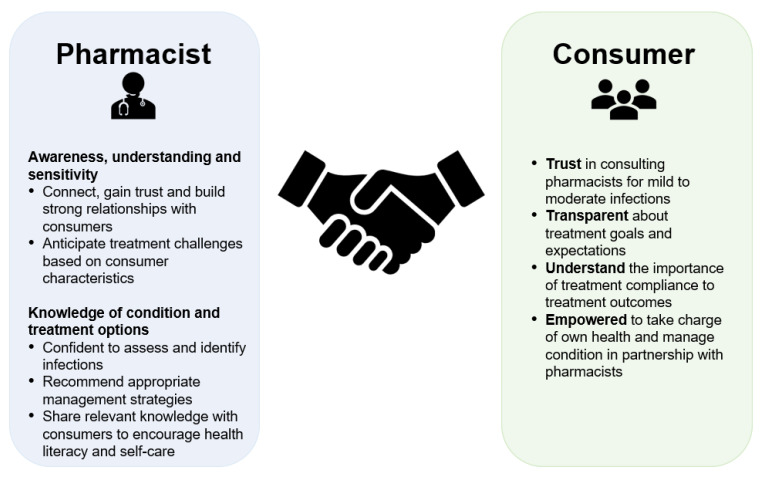
Key components of a successful care partnership between pharmacists and consumers for managing superficial fungal infections.

Overcoming challenges such as treatment compliance also requires a close partnership between pharmacists and consumers, with effective communication and alignment on concerns and expectations (Figure 5). The consumer survey showed that most consumers were not only bothered by the symptoms of SFI, but were also negatively impacted in terms of emotional and social wellbeing. In the pharmacist survey, relief of symptoms was indeed recognized as a top priority in SFI management; however, the consumer survey results show that it is also important for pharmacists to give attention to the emotional and social impact of SFIs during their interactions with consumers.

To address these gaps in consultation, the first two steps of the Fungal CARE guide (Categorize and Assess) encourage pharmacists to spend time probing for information that can help them develop a more tailored treatment approach for the consumer. The next two steps of the guide (*Recommend* and *Empower*) remind pharmacists to reach a mutual understanding with the consumer regarding treatment goals and share the knowledge necessary for successful treatment and preventing recurrence. This includes demonstrating proper application of medications and emphasizing the importance of treatment compliance. The use of the Fungal CARE guide will not only support pharmacists during SFI consultations, but will also help consumers feel more engaged to play an active role in their recovery and self-care.

## 6. Limitations

The authors acknowledge a number of limitations of this work. First, although the surveyed pharmacists clearly felt that the Fungal CARE guide would be useful in their practice, the framework has not yet been implemented and evaluated. Going forward, it will be important to evaluate the applicability of the framework, preferably in a range of community pharmacy settings, in order to determine where improvements and ancillary resources may be needed. Second, the pharmacist survey was a pilot survey that included 104 respondents. Although this number represents a small proportion of all registered pharmacists in Thailand [37], it should be noted that this was a pilot survey of relatively short duration (two months). Nevertheless, the majority of respondents were community pharmacists, matching the main target population for which the Fungal CARE guide was developed. The respondents included pharmacists practicing in rural as well as urban areas, and the major regions of the country (North, Northeast, and South) as well as the capital were represented. This suggests that the insights could be applicable across a range of community pharmacy settings. Findings from this pilot survey were presented in September 2021 in an educational webinar for pharmacists in Thailand. As a further follow-up alongside implementing and evaluating the Fungal CARE guide, a longer data collection period would be warranted to obtain feedback from a broader sample of pharmacists. Third, the consumer survey was conducted using a web-based online platform, and respondents were typically well-educated, of the middle socioeconomic class, and familiar with internet technology. Additionally, the selection criteria were for individuals aged 20–45 years (working-age adults) who had purchased and used a specific topical azole preparation (clotrimazole) for a recent fungal infection. Some of the findings may not be wholly generalizable to individuals outside of this age group (for example, children or older adults), or to those who require treatment beyond the indication of this type of topical azole. However, it is noted that working-age individuals often serve as caregivers for children or other family members with SFIs and thus may seek pharmacy services on behalf of these individuals. The demographic profile of the surveyed consumers also appears similar to that reported for typical users of community pharmacy services in other countries within the Association of Southeast Asian Nations (ASEAN) region [15,21,38]. Although the consumer and pharmacist surveys covered two different countries (Philippines and Thailand), the patterns of SFI etiology reported by consumers were similar and relatable to that described for other ASEAN countries [4].

## 7. Conclusions

Effective pharmacist-consumer consultations have great potential to improve management of SFIs by facilitating communication and alignment on key priorities and expectations for treatment. The Fungal CARE guide seeks to provide pharmacists with a structured yet collaborative patient-centered consultation process. Moving forward, the implementation, evaluation, and improvement of the Fungal CARE guide will be important to make it useful and practical for pharmacists to use in identifying and managing SFIs.

## Data Availability

The data discussed in this work are not publicly available but may be provided by the dataset owners upon reasonable request.

## Supplementary Materials

The following supporting information can be downloaded at: https://www.mdpi.com/article/10.3390/pharmacy10010029/s1, Supplementary File S1: Consumer Survey; Supplementary File S2: Pharmacist Survey.
