# Supplementary material for: Towards Better CARE for Superficial Fungal Infections: A Consultation Guide for the Community Pharmacy
Source: Pharmacy (Basel). 2022 Feb 11;10(1):29. doi: 10.3390/pharmacy10010029 (PMC8878117; doi:10.3390/pharmacy10010029)
Supplement: Supplementary file 1 [file pharmacy-10-00029-s001.zip › SupplementaryFile1_ConsumerSurvey.pdf]

Supplementary file 1. Consumer survey

Overview and survey methods

This was an online consumer satisfaction survey conducted nationwide in the Philippines from 31 March to 17 June 2020 by IQVIA Solutions Asia Pte Ltd on behalf of Bayer Philippines Inc. This consumer survey was conducted in accordance with the Code of Conduct of the UK Market Research Society. Respondents were recruited from an online consumer panel and were invited to participate in the online survey via a web link that directed them to a screening questionnaire. Respondents who met the screening criteria were invited to complete the full survey, and their responses were collected via a dedicated online platform. The target population for the survey was as follows: male or female consumers residing in the Philippines (any region), aged 20–45 years, who had used a topical antifungal (clotrimazole) in the past 12 months to treat their most recent fungal infection. A total of 500 male and female consumers who met the criteria were included and analyzed.

The survey questionnaire consisted of 14 Likert or multiple-option questions covering:

(i) Characteristics of the consumer’s most recent fungal infection

(ii) Symptoms, duration, and treatment of most recent fungal infection

(iii) Feelings and experiences related to the most recent fungal infection, including during treatment and recovery

(iv) Satisfaction and perceived benefits of the topical antifungal medication during treatment and recovery from the fungal infection

The survey responses were analyzed descriptively (tabulated and summarized as counts and percentages).
