# Supplementary material for: Towards Better CARE for Superficial Fungal Infections: A Consultation Guide for the Community Pharmacy
Source: Pharmacy (Basel). 2022 Feb 11;10(1):29. doi: 10.3390/pharmacy10010029 (PMC8878117; doi:10.3390/pharmacy10010029)
Supplement: Supplementary file 1 [file pharmacy-10-00029-s001.zip › SupplementaryFile2_PharmacistSurvey.pdf]

Supplementary file 2. Pharmacist survey

Overview and survey methods

This was an online pilot survey conducted in June-July 2021 among pharmacists working in Thailand. The survey was not limited to pharmacists practicing in specific professional roles, settings or healthcare facility types. Pharmacists were invited to participate in the survey via e-mail, online pharmacist network groups, social media, and quick response (QR) codes displayed in pharmacies, which directed them to the survey web page hosted on Google Forms. This landing page provided respondents with essential information on the purpose of the survey, and recorded their informed consent prior to completing the questionnaire. No personal data were collected. Respondents provided consent for their responses to be recorded anonymously and analyzed in aggregate.

The questionnaire consisted of 21 questions covering:

(i) General characteristics of respondents and their practice setting

(ii) Frequency and characteristics of SFI-related consultations

(iii) Current SFI management practice, including treatment options and selection considerations

(iv) Current perceived challenges in managing SFIs

(v) Views on the use of the Fungal CARE guide

The survey included a mixture of multiple-choice questions that allowed either single or multiple response options and ranking questions. At the end of the survey period, all responses were downloaded from Google Forms for analysis. Responses were analyzed descriptively (tabulated and summarized as counts and percentages). Findings from this survey were presented in September 2021 during an educational webinar for pharmacists in Thailand.
